# Statistical degradation modelling of Poly(D,L-lactide-co-glycolide) copolymers for bioscaffold applications

**DOI:** 10.1371/journal.pone.0204004

**Published:** 2018-10-01

**Authors:** Yaroslava Robles-Bykbaev, Javier Tarrío-Saavedra, Sara Quintana-Pita, Silvia Díaz-Prado, Francisco Javier García Sabán, Salvador Naya

**Affiliations:** 1 Instituto de Investigación Biomédica de A Coruña (INIBIC), Complexo Hospitalario Universitario de A Coruña (CHUAC), SERGAS, Departamento de Medicina, Universidade da Coruña, A Coruña, Spain; 2 GI-IATa, Universidad Politécnica Salesiana, Cuenca, Ecuador; 3 Grupo MODES, Departamento de Matemáticas, Escola Politécnica Superior, Universidade da Coruña, Ferrol, Spain; 4 Centro de Investigación TIC (CITIC), Universidade da Coruña, A Coruña, Spain; 5 DevelopBiosystem S.L., Cedeira, Spain; 6 ITMATI, Santiago de Compostela, Spain; University of South Carolina, UNITED STATES

## Abstract

This methodology permits to simulate the performance of different Poly(D,L-lactide-co-glycolide) copolymer formulations (PDLGA) in the human body, to identify the more influencing variables on hydrolytic degradation and, thus, to estimate biopolymer degradation level. The PDLGA characteristic degradation trends, caused by hydrolysis processes, have been studied to define their future biomedical applications as dental scaffolds. For this purpose, the mass loss, pH, glass transition temperature (*T*_*g*_) and absorbed water mass of the different biopolymers have been obtained from samples into a phosphate-buffered saline solution (PBS) with initial pH of 7.4, at 37°C (human body conditions). The mass loss has been defined as the variable that characterize the biopolymer degradation level. Its dependence relationship with respect to time, pH and biopolymer formulation has been modelled using statistical learning tools. Namely, generalized additive models (GAM) and nonlinear mixed-effects regression with logistic and asymptotic functions have been applied. GAM model provides information about the relevant variables and the parametric functions that relate mass loss, pH and time. Mixed effects are introduced to model and estimate the degradation properties, and to compare the PDLGA biopolymer populations. The degradation path for each polymer formulation has been estimated and compared with respect to the others for helping to use the proper polymer for each specific medical application, performing selection criteria. It was found that the mass loss differences in PDLGA copolymers are strongly related with the way the pH decay versus time, due to carboxylic acid groups formation. This may occur in those environments in which the degradation products remain relatively confined with the non degraded mass. This is the case emulated with the present experimental procedure. The results show that PDLGA polymers degradation degree, in terms of half life and degradation rate, is increasing when acid termination is included, when DL-lactide molar ratio is reduced, decreasing the midpoint viscosity, or when glycolide is not included.

## 1 Introduction

Biomaterials can be defined as those products safely implanted (permanently or temporary) in the body to simulate the tissue functions, to restore the existing effect and induce tissue regeneration [1]. Poly(D,L-lactic-co-glycolic acid), PDLGA, is an important synthetic [2] and biodegradable [3] biomaterial for many biomedical purposes. It is a copolymer based on DL-lactic and glicolyc acids used for different pharmaceutical and biomedical applications such as surgical implants, fixures, sutures, controlled drug delivery, and scaffolds in tissue engineering domain [4]. L-lactide and DL-lactide have been currently used for copolymerization with glycolic acid monomers, resulting the so called PLGA copolymers. PLGA biodegradable polymers have been commercially developed, obtaining materials with different mechanical properties [5]. They have been approved by the Food and Drug Administration for human use [6–9], being applied in locally implanted medical devices, scaffolds [8, 10], and as biodegradable carrier for the delivery [11, 12] of different therapeutic agents such as proteins, DNA, peptides, among others [12, 13]. In fact, the PLGA is the most extensively studied and used polymer for delivery applications [14], taking into account its biodegradability and biocompatibility properties [15]. As mentioned by Cristallini et al. [16], PLGA provides polymeric biomaterials where properties such as biocompatibility and biodegradation can be modulated by varying PLA and PGA fraction contents, among other advantages.

In this study, PDLGA polymers are tested and their in vitro behaviour is statistically modelled in order to evaluate their performance in tissue engineering applications. In situ tissue engineering is a increasingly important concept which involves engineering, cell and material science methodologies to act (replace or improve) over the body tissues. Among other applications, it accounts for the use of different materials and methods for covering bone defects, particularly critical. For example, in order to maintain alveolar processes after tooth extraction, where conventionally dentures or osseointegrated dental implants could be placed. In fact, after the loss of dental and periodontal tissue (due to different reasons) bio-scaffolding helps to promote new bone tissue growth, being used before placing a titanium implant [17]. In the present case, the PDLGA polymers which at the beginning were designed for developing drug delivery devices, now are studied to perform bioscaffolds. According to Wang [18], these scaffold materials specially applied in bone tissue engineering should meet the following 6 principles to perform optimally their functions: 1) the scaffold must be non-toxic and biocompatible, 2) biodegradable 3) highly porous and three-dimensional, 4) have the required mechanical properties to temporally provide structural support, 5) be processable to be modified in several sizes, 6) mimic the extra-celular matrix, and 7) osteoinductive and osteoconductive.

Reliability is one the branches of statistics with more applications in material engineering. In this work, estimating the degradation level of a material depending on time and other critical variables is intended. This has been tackled by the application of statistical reliability analysis techniques such as degradation models estimation. In fact, one of the applications of statistical degradation models in material engineering is the estimation of the material lifetime and reliability [19]. In this context, while lifetime is the duration after which a material cannot fulfill its design requirements [20], reliability is technically defined as the probability that a material performs its function under work conditions, during an interval of time [19]. In some reliability studies, as in the present case, it is possible to measure physical degradation (mass loss, water absorption, pH) depending on time. This type of data are named degradation data. Degradation is strongly connected with aging, that is the time dependent process defined by the decay of material critical to quality (CQT) design properties as mass, modulus and other mechanical characteristics [20]. This aging can be produced by the action of pressure, water, mechanical stress, heat, among other causes [21, 22]. It is important to note that the proper statistical degradation modelling and accurate lifetime estimates of the biopolymers used as bioscaffolds are crucial for an adequate material selection, according to the application requirements. These types of studies can be helpful for continuously performing and designing better scaffolds, customized depending on the application [23]. In fact, there are some applied mathematics, including kinetics, computer aided, and statistical works devoted to the scaffold degradation and lifetime modelling [24–28], where degradation models for CTQ variables are performed from very different approaches. Chen el al. [24] developed a numerical model taking into account the stochastic hydrolysis and mass transport to simulate the polymeric degradation and erosion process. Hoque et al. [25] also modelled the mass loss using an exponential expression, assuming the water diffusion and hydrolysis as the main causes for the degradation processes. Focusing on lactic and glycolic based polymers, pH-dependant kinetics models have been applied to explain the hydrolytic degradation of lactic acid [29] and D,L-lactic acid oligomers [30]. Kinetic models based on logistic function have been also applied to estimate the PDLA polymers lifetime due to thermal aging [21]. Moreover, Idaszek et al. [31] have described the hydrolytic degradation of PLGA, and they have identified the more influencing variables using statistical exploratory analysis and linear regression. The mechanical and viscoelastic properties are also CTQ variables to measure the extend of biopolymer degradation. Accordingly, the work of Zhang [23] presents a review of mechanical and physical properties of biodegradable polymers, whereas Breche [32] proposes a physically motivated model to estimate the mechanical behaviour of PLA-b-PEG-b-PLA copolymers during hydrolytic degradation.

The present work focuses on the degradation study (in vitro) of PDLGA polymers and copolymers due to hydrolysis under human body conditions. Thus, it is important to note that only the degradation due hydrolysis is modelled. As pointed out by Breche [32], to obtain a comprehensive knowledge about the evolution of scaffold properties during degradation is necessary. In fact, we have to ensure that the scaffolds adequately replace the functions of tissues. It is also important to note that modelling can be useful to minimize the amount of experiments needed for characterizing a polymer library [33]. This can be tacked by applying statistical modelling, taking into account that polymer degradation is depended on multiple factors whose influence have to be accurately determined [34, 35]. This study is useful for tasks such as estimating the mass loss of PDLGA biopolymers during application, depending on time and pH, under human body conditions. The results could help to choose the proper PDLGA formulation depending on each medical application. In the case of bioscaffolds, the PDLGA formulation could be chosen in order to balance the bone tissue cell growth and the biopolymers mass loss. In addition, the PDLGA mass loss dependence of pH is studied. A strong statistical relationship between these variables can help the practitioners to estimate the degradation trend knowing the pH value and vice versa (in those case where the material is relatively confined with the degradation products).

For these purposes, statistical learning (field referred to statistics and computer science interrelation) tools for complex datasets have been applied to model the degradation trends and reliability of the studied materials [19, 36], previously identifying the most influencing variables on PDLGA degradation.

This proposal is structured as follows: Section 2 describes the materials, sample preparation, testing and scaffold characterization. In Section 3, we introduce the Generalized Additive Models (GAM) and parametric nonlinear mixed-effects regression applied to describe the PDLGA degradation. Section 4 accounts for the results (and the corresponding discussion) of statistical modelling applied to PDLGA degradation data. Finally, in Section 5 the main conclusions of the present study are stated.

## 2 Experimental

### 2.1 Materials

**Fig 1:**
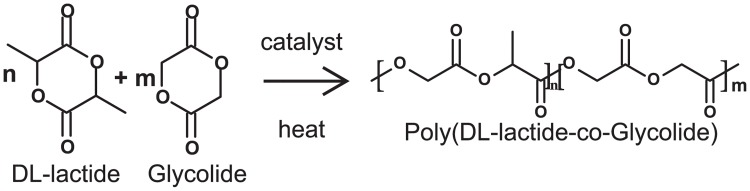
Biopolymers formulation based on PLA and glycolic acid (Courtesy: PURAC-CORBION, 2010).

PDLGA, such as other biomaterials used for biomedical purposes, should be nontoxic (glycolic and lactic acids are easy metabolized by the body) [37], bioresorbable, able to attach the cells, promote their proliferation and differentiation, and also degrade according to physiological rates [38, 39]. Thus, it is crucial to estimate the degree and rate of degradation of this type of materials, in order to apply the proper material depending of the application. That is also the case of PDLGA formulations, based on different molar ratio proportions of DL-lactide an glycolide, studied in this work (Fig 1). Table 1 shown all the different PDLGA formulations, provided by PURAC-CORBION, with their molecular weight. Each copolymer tag provides information about composition (including or not glycolide), the DL-lactide molar ratio, the viscosity midpoint value, and if it is or not acid terminated. For example, PDLG7502A is a copolymer composed of DL-lactide and glyolide, with a DL-lactide molar ratio of about 75%, a viscosity midpoint of 0.2 dl/g, and acid terminated (A).

**Table 1:** PDLA polymers tested and modelled in this study. The tag, proportion of DL-lactide and glycolide, and molecular weight are provided. The symbol * means that is relative to polystyrene standards (indicative values based on a Mark Houwink type correlation).

TAG	POLYMER	Mw* [Kg mol^−1^]
PDLG 5002	50/50 DL-lactide/glycolide copolymer	17
PDLG 5004	50/50 DL-lactide/glycolide copolymer	44
PDLG 5010	50/50 DL-lactideh/glycolide copolymer	153
PDLG 7502	75/25 DL-lactide/glycolide copolymer	17
PDLG 7502A	75/25 DL-lactide/glycolide copolymer	17
PDLG 02A	Poly(DL-lactide)	17
PDLG 02	Poly(DL-lactide)	17

### 2.2 Sample preparation and testing

**Fig 2:**
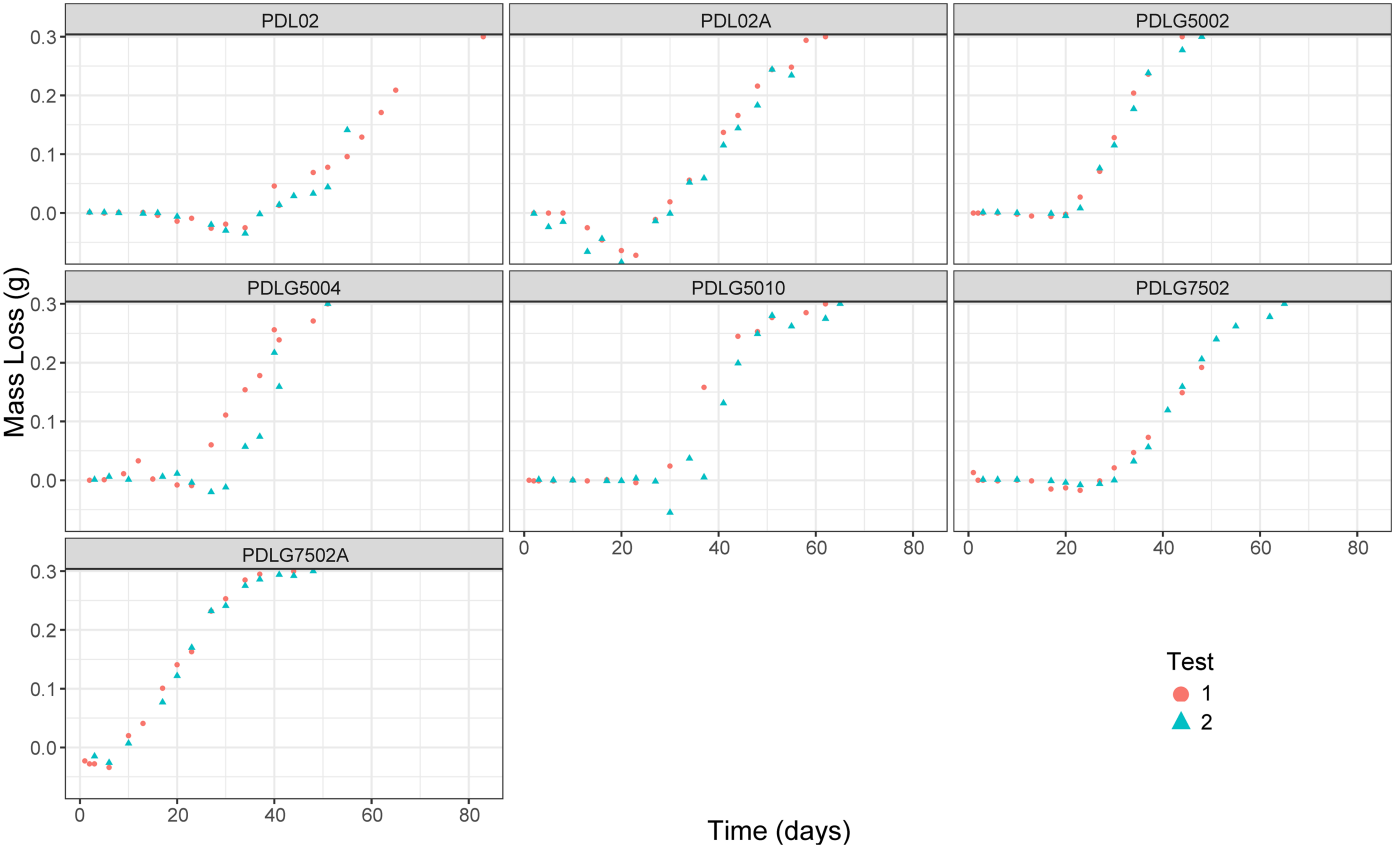
The two degradation paths (two replicates per polymer have been performed in laboratory) corresponding to each polymer are shown. They are obtained by experimental procedures in lab.

The sample preparation scheme has been designed in order to obtain the degradation path due to hydrolysis of the different PDLG formulations. All the samples have been maintained at 37°C and the initial pH has been 7.4, values in the range of human body conditions. The samples tested by each polymer formulation are 28 for the PDLG5002, 27 for PDLG7502A, 33 of the PDLG7502, 34 of the PDLG5010, 30 of PDLG5004, 36 of PDL02, and 33 of PDL02A. It is important to note that two degradation paths have been obtained for each PDLGA polymer. Namely, in the case of PDLG5002, the first degradation path (first replicate) is composed of 14 samples. They are collected and prepared (see description below) at the same time, separately. Each about 3 days, one sample is extracted from the bath and the mass loss, pH and other critical variables for understanding the degree of polymer degradation are obtained. Thus, each sample provides information of one point of the degradation trajectory (at each time a mass loss is assigned). After obtaining the first degradation trajectory, another experiment is developed, the second replicate: other 14 samples of PDLG5002 have been prepared and, following the same procedure, 14 pairs (time, mass loss) have been obtained. These 14 pairs accounts for the second degradation path (the second replicate) of the PDLG5002. The Fig 2 shows the two degradation trajectories for the PDLG5002 and also for the remaining PDLGA formulations.

**Fig 3:**
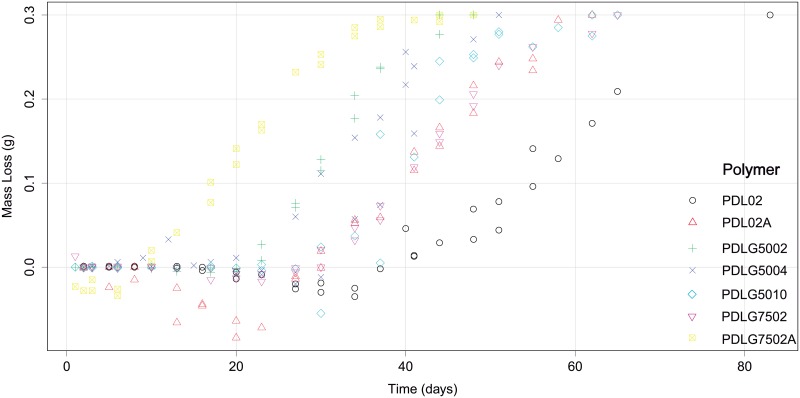
Experimental data of mass loss vs time corresponding to the 7 different polymers. Note that the two degradation trajectories corresponding to each polymer are shown in the same colour.

In the case of the PDLG7502A, 14 samples correspond to the first degradation path, whereas 13 account for the second degradation trajectory. With respect to the PDLG7502, the first replicate is composed of 15 samples, whereas the second degradation trajectory has 18. In the remaining polymers, first and second degradation paths are composed of 15 and 19 (for PDLG5010), 15 and 15 (for PDLG5004), 20 and 16 (for PDL02), and 18 and 15 (for PDL02A) samples, respectively. The two degradation paths obtained for each polymer are shown and identified in the Fig 2. In addition, The Fig 3 provides information about the differences between PDLGA polymers taking into account their degradation paths (note that the two degradation trajectories corresponding to each polymer are depicted here in the same colour).

The information of each sample of biopolymer is obtained using the following procedure. Each vial is filled with 0.3 g of biopolymer and 1 ml of phosphate-buffered saline solution (PBS), with an initial pH of 7.4. Then, they are closed and placed in a bath at 37°C. After about 3 days, each vial is taken out of the bath, the saline solution is removed and the pH of the solution is measured. After removing the saline solution, the vial is weighed again, obtaining the wet sample weight and the percentage of the liquid that has been absorbed by the polymer (% H_2_O gain). Subsequently, each vial is dried into an oven at 37-40°C during 24. Once the sample has been dried, the vial and the polymer are weighted again together and the final dry sample weight is obtained. The degradation of these polymers is studied until they completely lost their mass. Moreover, the glass transition temperature of each polymer sample is estimated by Differential Scanning Calorimetry (DSC). Once all the weights are obtained, samples of 5 mg are removed, and then heated from 0 to 100°C at 10°C min^−1^, under a N_2_ flow of 50 ml min^−1^, in a TA Q2000 instrument.

## 3 Statistical modelling

Once experimental degradation data at 37°C are obtained, applying statistical techniques is necessary in order to characterize and model the degradation paths due to hydrolysis of PDLGA formulations. First of all, using graphical tools of data mining exploratory analysis such as scatterplot matrices is necessary to extract valuable information concerning with polymer degradation from the multivariate dataset. Statistical descriptive analysis allows to study the dependence relationships between variables, whether due to real causality or not, and to identify the critical variables for hydrolytic degradation. Afterward, the dependence modelling between critical variables can be performed by the estimation of nonparametric models such as GAM models [40], and parametric nonlinear regression models of fixed and mixed effects. The aim is to estimate the more influencing variables over polymer mass loss, the functions which relate them, and their parameters to define the path of degradation of each PDLGA formulation, and help to develop selection criteria depending of the scaffold application.

### 3.1 Generalized additive models (GAM)

GAM modelling is an extension of the Generalized Linear Models [40] that are extensively used in many science an technology domains [41–44]. The response variable can be Gaussian, Gamma, Poison, or Binomial distributed and it is expressed as a function of one or more explanatory variables (quantitative and qualitative). The goal is to estimate the explained variable assuming that the effects of continuous covariates are unknown but “soft”. This model provides a flexible estimation of the response variable using smothers such as the penalized regression splines, among other alternatives. Covariates, factors and interactions can be included (as smooth effects and/or as linear function) in this model. In this regard, the expression 1 depicts a GAM model where *η* is the mean of *Y* response variable, *g* a link function (it depends on the *Y* probability distribution, identity matrix for Gaussian case), *X*_1_ and *X*_2_ are independent variables (covariates or factors) whose effects on *Y* are linear, whereas *T*_1_ and *T*_2_ are scalar explanatory variables whose effect over the response are nonparametric and subjected to smoothing process, being *f*_1_(*T*_1_) the smooth effect of *T*_1_ over response *Y*, and *f*_12_(*T*_1_, *T*_2_) the smooth effect of *T*_1_ and *T*_2_ interaction.

$$\begin{array}{c} g\left(\eta \right)=\beta_{0}+\beta_{1}X_{1}+\beta_{2}X_{2}+f_{1}\left(T_{1}\right)+f_{2}\left(T_{2}\right)+f_{12}\left(T_{1},T_{2}\right) \end{array}$$ (1)

In a more formal manner, the quantitative response variable *Y* is the polymer mass loss due to hydrolysis can be estimated by a sum of linear functions of variables contained in ***X*** matrix and *k* smooth functions of the covariates *T*_*j*_, according to the expression:

$$E\left(y\right)=\mu =g^{-1}\left(\beta X+\sum_{j=1}^{k}f_{j}\left(T_{j}\right)\right),$$

with *X*_*i*_ the columns of ***X***, *i* = 1, …, *m*.

In the present case, the covariates *T*_*j*_, with *j* = 1, 2, are the time and pH, respectively, and **X** accounts for the polymer formulation variable. Thus, *β* parameters are the linear effect parameters (corresponding to each class of polymer), and *f*_*j*_(*T*_*j*_) are the smooth functions of time and pH, expressed as a function of *b*-splines basis. Estimates of *β*, $\hat{\beta }$, and *f*_*j*_, ${\hat{f}}_{j}$, are obtained from the experimental sample using the Backfitting algorithm [40].

GAM modelling can be defined without assuming a parametric dependence relationship between response and explanatory variables. Therefore, this technique can be useful to determine the underlying parametric models that define these relationships by observing the estimated smooth effects of explanatory variables over the response, *f*(*T*_*i*_).

### 3.2 Parametric regression techniques

The use of parametric modelling is needed in this study due to we search for obtaining the function defined by a set of parameters with physical–chemical meaning that describes the path of degradation corresponding to PDLGA polymers and characterizes them [45, 46]. This can not be done from a nonparametric point of view.

The expression 2 shows a simple regression model structure:

$$\begin{array}{c} y_{i}=m\left(t_{i}\right)+\varepsilon_{i},\;\;i=1,2,\ldots ,n,\;\;\;with\;E\left(\varepsilon_{i}\right)=0, \end{array}$$ (2)

whereby *m* is the regression function that provide the values of the response variable *Y* as a function of the independent variable *T*, and *ε*_*i*_ are zero mean independent and identical distributed residuals, satisfying 0 ≤ *t*_1_ < *t*_2_ < ⋯ < *t_n_* < 1 with *n* the number of observations. If *m* is parametric, we define *M* = {*m*_*θ*_(•)/*θ* ∈ Θ}, whereby *θ* is the parameter vector that defines the model, whereas Θ is a subset of $R^{k}$.

**Fig 4:**
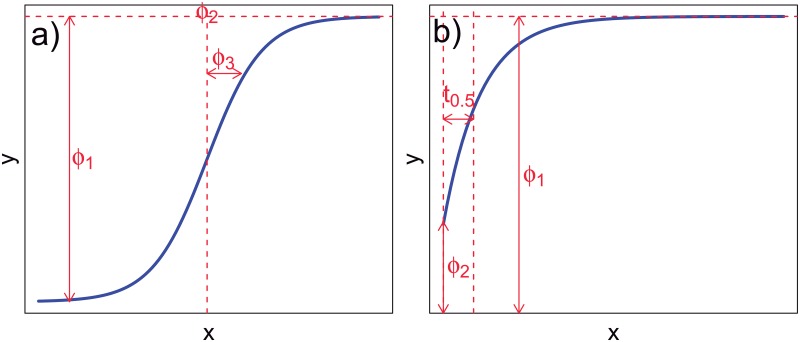
Shape and parameters of logistic (a) and asymptotic (b) functions.

In the present work, the relationships between critical degradation variables are non linear. In fact, it is shown that they can be described by asymptotic and logistic nonlinear functions. The logistic function can be expressed as

$$\begin{array}{c} y=\phi_{1}+\frac{\phi_{2}-\phi_{1}}{1+\exp(\frac{x-\phi_{3}}{\phi_{4}})}, \end{array}$$ (3)

whereby *ϕ*_1_ accounts for the estimated initial value of *Y* (response variable), *ϕ*_2_ is the estimated final value of *Y*, *ϕ*_3_ the value of *x* (independent variable) at the inflexion point of the sigmoid curve, and *ϕ*_4_ is the scale parameter, regarding the change rate of the function (when *x* = *ϕ*_3_ + *ϕ*_4_, the response is broadly three-quarters of the distance from *ϕ*_1_ to *ϕ*_2_). On the other hand, the asymptotic function follows the expression

$$\begin{array}{c} y=\phi_{1}+\left(\phi_{2}-\phi_{1}\right)\;\text{exp}\left(-\text{exp}\left(\phi_{3}\right)x\right), \end{array}$$ (4)

where *ϕ*_1_ is the horizontal asymptote pointing out the initial value of *Y* estimates, *ϕ*_2_ the value for *Y* corresponding to *x* = 0 (pH in the present case), and *ϕ*_3_ is the natural logarithm of the rate constant (and the half-life *t*_0.5_ = log2/exp(*ϕ*_3_)). Fig 4 shows the shape and graphical meaning of the parameters corresponding to asymptotic and logistic functions.

#### 3.2.1 Nonlinear mixed-effects regression models

It is important to stress that, in addition to nonlinear relationships, the effects over the response (mass loss) corresponding to, on the one hand, the type of polymer factor and, on the other hand, the replication (two tests are performed) factor, have to be also taking into account in the modelling task. In fact, this is a nonlinear mixed-effect regression problem of repeated measurements, assuming that 2 different tests are developed for each type of commercial PDLGA polymer.

The application of mixed-effects regression models is recommended when describing the relationship of a response variable with respect a number of covariates is required in the framework of grouped data (data depended on one or more classification factors) [19, 45, 47–49]. In this way, fixed effects model parameters accounts for the information of the whole population, whereas parameters random effects are associated with individual experimental units sampled from the entire population. Taking into account these premises, mixed-effect regression models are defined by parameters with both fixed and random effects, unlike standard linear an nonlinear regression where only fixed effects are assumed. As pointed out by Pinheiro and Bates [47], nonlinear mixed-effects models are those mixed-effects models in which at least one of the fixed and random effects is nonlinear in the model function. In this case study, considering that degradation data depend on PDLGA formulation and replicate factors (grouped data), the application of nonlinear regression models with mixed effects is useful and necessary to estimate the nonlinear parametric relationship (sigmoid or asymptotic) between mass loss and time (alternatively, mass loss depending on pH, and pH versus time). In addition, the variability of response variable corresponding to PDLGA polymer formulation and replicate factors can be estimated separately from the residual error. In a mixed-effects model, the random effects accounts for the deviations of individuals from the mean value of the parameters (fixed effects) that represent the population, in this case, PDLGA formulation and replicate. Summarizing, nonlinear mixed-effects models are preferentially used when (1) dealing with grouped data, where (2) individuals are samples of a studied population, (3) providing factor variability estimates, (4) through more parsimonious (less number of parameters) expressions than the corresponding to fixed effects counterparts. They are just defined to deal with grouped data, sharing the main goals of nonlinear regression when compared with linear or polynomial regression: parsimony, physical interpretability of model parameters and validity of predictions beyond the experimental data range [47].

Accordingly, mixed-effect regression models based on logistic and asymptotic functions are proposed in this work. Assuming any nonlinear function *f* depending on *ϕ* vector parameters and *v* covariates, the nonlinear mixed-effects models for two nested factors (two levels of nesting) can be parametrized as follows [47]:

$$\begin{array}{c} y_{ijk}=f\left(\phi_{ijk},v_{ijk}\right)+\varepsilon_{ijk},\\ i=1,\ldots ,M,j=1,\ldots ,M_{i};k=1,\ldots ,n_{ij}, \end{array}$$ (5)

whereby *y*_*ijk*_ are the values of the response variable, *M* denotes the number of first-level different groups (in this case, PDLGA formulation factor), *M*_*i*_ is the number of second-level groups (replicate factor) within the *ith* group of first-level, *n*_*ij*_ is the number of observations within each *jth* second-level group corresponding to the *ith* group of the first-level, and *ε*_*ijk*_ is the within-group error term (normally and independently distributed as $\varepsilon_{ijk}∼N\left(0,\sigma \right)$, independent of the random effects). Moreover, *f* accounts for a differentiable nonlinear (at least in some *ϕ*_*ij*_) real-valued function defined, for a specific group, trough a parameter vector *ϕ*_*ijk*_ and a covariate vector *v*_*ijk*_. The parameter vector can be also defined by

$$\begin{array}{c} y_{ijk}=A_{ijk}\beta +B_{i,jk}b_{i}+B_{ijk}b_{ij}+\varepsilon_{ijk},\\ b_{i}\sim N(0,\Psi_{1}),b_{ij}\sim N(0,\Psi_{2}) \end{array}$$ (6)

whereby *β* depicts a fixed effects *p*-dimensional vector, being **A**_*ijk*_ the corresponding design matrix (depend on data and model), *b*_*i*_ are the *q*_1_-dimensional vectors of first-level random effects, normally and independently distributed with Ψ_1_ variance-covariance matrix, *b*_*ij*_ are the *q*_2_-dimensional vector of second-level random effects, also normally and independently distributed with Ψ_2_ variance-covariance matrix, and **B**_*i*,*jk*_ and **B**_*ijk*_ are the random effects design matrices that depend on grouping first and second level. The model parameters are estimated by maximum likelihood, based on the marginal density of the responses, as pointed out in [47].

It is important to note that data analysis tasks have been performed using statistical software R. In this regard, the functions of mgcv package have been used to estimate GAM models, whereas the nlme library [50] has been used to estimate nonlinear mixed-effects regression models.

## 4 Results and discussion

This section accounts for the main results (and their companion discussion) of exploratory and modelling statistical techniques application to degradation data described in section 2. The critical variables that best describe the hydrolytic degradation process of the studied PDLGA polymers are identified. Their dependence relationships are defined through statistical modelling and, using the models estimates, the different PDLGA formulations are compared to help to develop selection criteria in dental scaffolding and other medical applications.

### 4.1 Exploratory analysis and nonparametric modelling (GAM)

**Fig 5:**
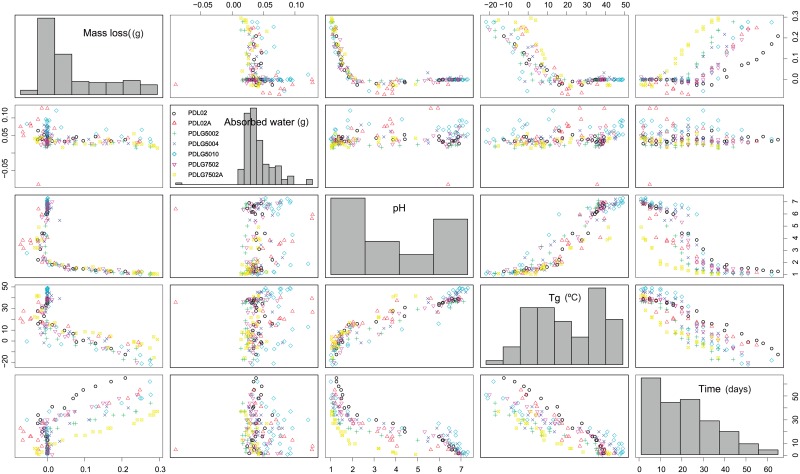
Descriptive analysis of the dependency structure between biopolymers.

The first step of every statistical analysis is to properly describe the data sample. Taking into account that almost all the obtained random variables that characterize the PDLGA samples are quantitative, one of the best options to obtain exploratory information about their distribution (range and frequency of values) and the pairwise relationships is to develop a scatterplot matrix. In addition, this type of graphical technique allows to include the effect of factors such as PDLGA formulation. Accordingly, Fig 5 shows the scatterplot matrix (with histograms for each variable) obtained from the studied variables that could account for PDLGA degradation information: mass loss (g), absorbed water mass (g), pH, glass transition temperature (*T*_*g*_, measured in °C), time, and PDLGA formulation included as a factor or qualitative variable. Considering the mass loss the characteristic that more intuitively describe the degree of degradation, the pairwise relationships between polymer mass loss and the remaining variables are mainly observed. When mass loss variable is compared with polymer pH, a strong nonlinear asymptotic type trend is obtained (Fig 5). When pH increases, the mass loss also decreases in a nonlinear manner. It is highlighted that the mass loss seems to vary in the same way with respect to pH independently of the PDLGA formulation. A sigmoid nonlinear relationship between mass loss and time is also observed. Unlike the mass loss-pH relation (only one trend), a different well defined sigmoid trend is observed for each PDLGA polymer (panel in the first row and fifth column of the scatterplot matrix). Thus, it seems that the polymer mass loss strongly depends on time and PDLGA formulation. All the trends seem to be monotonically increasing. A greater amount of polymer is lost at longer times of degradation, observing a nonlinear trend between an initial (at zero mass loss) and final (at 0.3 g) asymptote. This fact support the use of further modelling tools based on sigmoid functions such as logistic. In addition, a different decreasing sigmoid relationship between mass loss and *T*_*g*_ (obtained by DSC) is observed for each PDLGA polymer. Higher *T*_*g*_ corresponds with lower amount of mass loss. It seems to be an initial asymptote concerning high levels of mass loss and low *T*_*g*_ and a final asymptote regarding with no mass loss and high *T*_*g*_. Between the two asymptotes there is a nonlinear relationship, with increasing rate of change until an inflexion point from which the slope continuously decreases. This fact supports that glass transition temperature is strongly related with the PDLGA mass loss and, thus, the level of hydrolytic degradation.

The previous analysis has been done just using the sample data and discussions are only valid in the case of the studied sample of PDLGA polymers. Obtaining models that properly define these relationships, not only at sample level but for the entire population (population of different PDLGA formulations), is intended. In addition, we search for ensuring if nonlinear trends detected in exploratory analysis are appropriate to define the relationship between critical variables at a population level. Nonparametric models are useful to obtain information about the dependence structure. Moreover, considering there are more than two critical variables, using regression models that allow us to deal with multivariate data structure is necessary. This is the case of GAM models (Section 2). Therefore, GAM models based on b-splines basis smoothing are estimated from sample data in order to obtain population estimates. The response variable, critical feature for polymer degradation, is the polymer mass loss, whereas pH (quantitative), time (quantitative), *T*_*g*_ (quantitative) and PDLGA formulation (qualitative) are the independent variables.

Considering the information obtained from Fig 5, three GAM models are proposed: the first one, GAM1, to estimate the mass loss as a function of time and PDLGA formulation, *E*(*Mass loss*) = *β*_0_ + *β*_1_*PDL*02*A* + *β*_2_*PDLG*5002 + *β*_3_*PDLG*5004 + *β*_4_*PDLG*5010 + *β*_5_*PDLG*7502 + *β*_6_*PDLG*7502*A* + *f*_1_(*Time*); the second, GAM2, to model the mass loss depending only of pH, *E*(*Mass loss*) = *β*_0_ + *f*_1_(*pH*); and the third, GAM3, to express the mass loss with respect to *T*_*g*_ and polymer formulation, *E*(*Mass loss*) = *β*_0_ + *β*_1_*PDL*02*A* + *β*_2_*PDLG*5002 + *β*_3_*PDLG*5004 + *β*_4_*PDLG*5010 + *β*_5_*PDLG*7502 + *β*_6_*PDLG*7502*A* + *f*_1_(*T*_*g*_). In order to develop the models, the dichotomous variables PDL02A, PDLG5002, PDLG5004, PDLG5010, PDLG7502, and PDLG7502A are defined. Their values are 1 when the corresponding polymer is studied and 0 in the remaining cases. In addition, only smooth effects over the response have been considered for all the quantitative variables, while the response is assumed Gaussian distributed.

The *X* predictors have been chosen taking into account their dependence structure. There is not recommended to include covariates that are depended, i.e. that could explain the same information about the *Y* response variable. In fact, the estimation of a GAM model with Mass Loss as response variable and pH, Time and T_*g*_ as predictors is not recommended in the present case due to the strong dependence relationship between these three last covariates. Consequently, if they are included together in the model, their corresponding effects on the response variable would not be reliably estimated. Indeed, the estimates of the effect of each predictor would be confused with the other effects. In the case of GAM2, the PDLGA formulation has been not included due to the linear effects of each polymer formulation are not significant different from zero (p-values>0.05). It seems that Mass Loss depending on pH is the same for all the PDLGA formulations. This result will be commented below, when more evidences be obtained using regression modelling.

**Fig 6:**
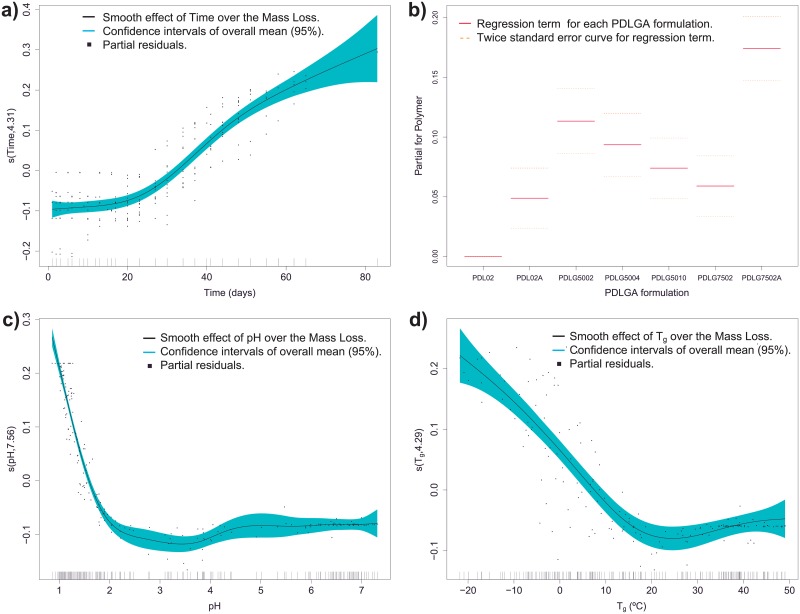
Smooth effects of time (panel a), and PDLGA formulation (panel b) over mass loss response corresponding to GAM1 model. Smooth function of pH when estimating mass loss (panel c) with the GAM2 model. Smooth effect of *T*_*g*_ over the mass loss response (panel d) of GAM3 model.

When first GAM model is performed, GAM1, the smooth effect of time and the partial linear effect of PDLGA formulation explain the 80.2% of mass loss overall variability (adjusted *R*^2^ = 0.802 and explained deviance of 81.2%). Fig 6a and 6b shows the smooth effect of time and the partial effect of PDLGA formulation, respectively. The smooth effects are labeled as *s*(*covariate* *name*, *smoothing* *degrees* *of* *freedom*). At this point, it is important to note that the smooths in a GAM model are centred in order to ensure model identifiability, i.e. each *s* is estimated taking into account the constraint that ∑_*i*_ *s*(*x*_*i*_) = 0, where *x*_*i*_ are the covariate values. The effect of time over the response is a growth sigmoid type curve (longer times correspond to higher mass loss), and it is significant different from zero. All the parameters corresponding to PDLGA formulation are also significant different from zero (*p*-values for all the *β*_*i*_ with *i* = 0, …, 6 are lower than 0.05). Fig 6b shows the effects of each PDLGA polymer with 2 × standard error tolerance, fixing at zero the parameter corresponding to PDL02 polymer, as a reference. It is observed that the existence of acid termination in polymers induces significant higher mass loss (e.g. PDL02 and PDL02A). Moreover, for an increasing midpoint viscosity the mass loss decreases, it seems that the polymer formulation is more stable against hydrolytic degradation (see the effects of PDLG5002, PDLG5004, and PDLG5010 in Fig 6b). It is also observed that, regarding PDLGA polymers with the same viscosity midpoint (and without acid termination), the formulations with higher DL-lactide molar ratio tend to loss a less amount of mass in the same interval of time, i.e. they are more stable against the hydrolytic degradation (see the effects of PDLG5002 and PDLG7502 in Fig 6b). In this case, all the factors are constant excepting DL-lactide molar ratio, thus, the latter could be an influencing variable over polymer degradation stability (measured by the polymer mass loss). Could be also interesting to study the effect of molecular weight on mass loss, but there are only three different values (153 Kg mol^−1^ for PDLG5010, 44 for PDLG5004 and 17 for the remaining, see Table 1) resulting on a non significant effect over the response. In fact, if Fig 6b is observed, no clear relationship between molecular weight and mass loss is shown. In order to test properly the effect of molecular weight, should be necessary to extend the analysis to other polymer formulations in the framework of further studies. Regarding the GAM2 model, the 93.9% (adjusted *R*^2^ = 0.939 and explained deviance of 94.1%) of mass loss variability is explained by only the smooth effect of pH, which means that polymer mass loss is very strongly depended of the polymer pH. Consequently, the mass loss of polymer could be estimated using only the pH as independent variable. In addition, Fig 6c shows that the pH smooth effect could be an asymptotic function as pointed out in the descriptive analysis. With respect to GAM3 model, the 76.8% (adjusted *R*^2^ = 0.768 and explained deviance of 78.2%) of mass loss variability is explained by the smooth effect of *T*_*g*_ and the partial regression effect of PDLGA formulation. If only *T*_*g*_ smooth effect is introduced in the model, the *R*^2^ = 70%. Thus, the relationship between mass loss and *T*_*g*_ is significant but weaker than the corresponding to mass loss-pH and mass loss-time. Moreover, it is important to stress that the *T*_*g*_ smooth effect could be assumed decreasing sigmoid, as suggested after descriptive analysis. As in the case of pH, the value o *T*_*g*_ has relevant information about the degradation degree of a PDLGA polymer.

Summarizing, GAM modelling supports the results of exploratory analysis at population level, i.e. the relationship between mass loss and time could be sigmoid, the effect of pH over the mass loss is asymptotic, the effect of the PDLGA formulation is significant different from zero and different depending on the formulation. It also important to note that the relationship between mass loss and pH is very strong allowing us to obtain reliable degradation degree predictions using GAM models. Alike, GAM models as a function of time and PDLGA formulation also could determine the polymer mass loss. Taking into account these results, to develop parametric models with physical-chemical meaning that could help to compare different formulations in addition to predict their degree of degradation is possible and useful.

### 4.2 Modelling of PDLGA degradation by nonlinear mixed-effects regression

The use of parametric regression models is proposed to estimate the PDLGA degree of degradation. The advantage of using parametric regression is to provide parametric expressions that link the degradation critical variables, depending on parameters with physical-chemical meaning. Among other goals, this type of regression is able to provide more exact and accurate estimates beyond the range of experimental data than many nonparametric models [47]. Among all the modelling options, considering the results of the previous section and the fact that the sample data are grouped, nonlinear mixed-effect models are proposed. Indeed, just a sample of 7 out of all the possible PDLGA formulations are studied and, testing just two replicates for each one. Therefore, it is recommended to use mixed-effects regression models, indeed it is better to estimate the model for the population that also allows to estimate variability due to PDLGA polymer and test (replicate) factors separately from the residual variance (mixed effects) than estimating a model for each trend (only fixed effects). Moreover, mixed-effects models are more parsimonious than fixed effect counterpart. Accordingly, the mean values of the parameters corresponding to the entire population of PDLGA formulations (including replicates) can be estimated through parameter fixed effects. In addition, as different trends with respect to the different levels of PDLGA and replication factors are expected, the population variance corresponding to these factors can be estimated by nonlinear mixed-effects models (trough parameters random effects).

**Fig 7:**
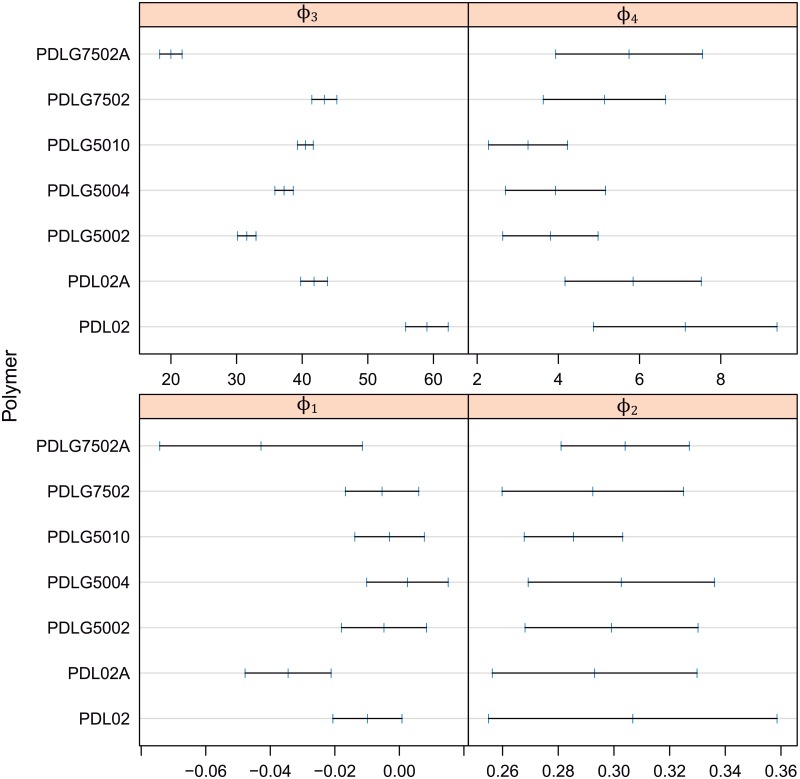
Confidence intervals (at 95% confidence level) for the parameters of the nonlinear model of mass loss versus time an PDLGA formulation, with only fixed effects. Parameters intervals are obtained from the 2 × 7 fittings.

The results obtained in the Section 4.1 support the modelling of mass loss versus time and PDLGA type and, in addition, the mass loss as a function of pH using nonlinear mixed-effects models. Considering an intuitive start-point, the first step is to estimate the best model for polymer mass loss as a function of time and PDLGA formulation. This is a more common case of degradation data where the critical to quality variable (mass loss) depend on time. As observed in section 4.1, the logistic function (Eq 3) is an adequate option to model the relationship between mass loss and time. Thus, firstly, nonlinear fixed effects models based on logistic functions are fitted and the 95% confidence intervals for the logistic parameters are estimated (from the 2 × 7 curves) and shown in Fig 7 for each PDLGA polymer. As pointed out in Fig 7, the differences between polymers are more significant if *ϕ*_3_ and *ϕ*_4_ parameters are observed, in the other cases the intervals are more overlapped. The differences in nonlinear trends due to PDLGA type are concerning with the inflexion point of logistic curve (half life time) and the degradation rate parameter. Therefore, in order to obtain a more parsimonious model, random effects due to PDLGA type are added only to *ϕ*_3_ and *ϕ*_4_ parameters obtaining a nonlinear mixed-effects model. Nested in the PDLGA factor, random effects due to test or replicate are also introduced in *ϕ*_3_ and *ϕ*_4_ parameters in order to estimate the variability due to replication separately of residual variance. In addition, the *ϕ*_1_ and *ϕ*_2_ parameters are fixed to 0 and 0.3 g, respectively, taking into account the initial mass loss of each experiment is 0 (absence of degradation) and the final asymptote has to coincide with the mass of each specimen, 0.3 g (concerning the total degradation of PDLGA samples). In fact, 0 and 0.3 values are within the parameter intervals for *ϕ*_1_ and *ϕ*_2_, respectively. As a result, only *ϕ*_3_ and *ϕ*_4_ parameters have to be estimated.

**Fig 8:**
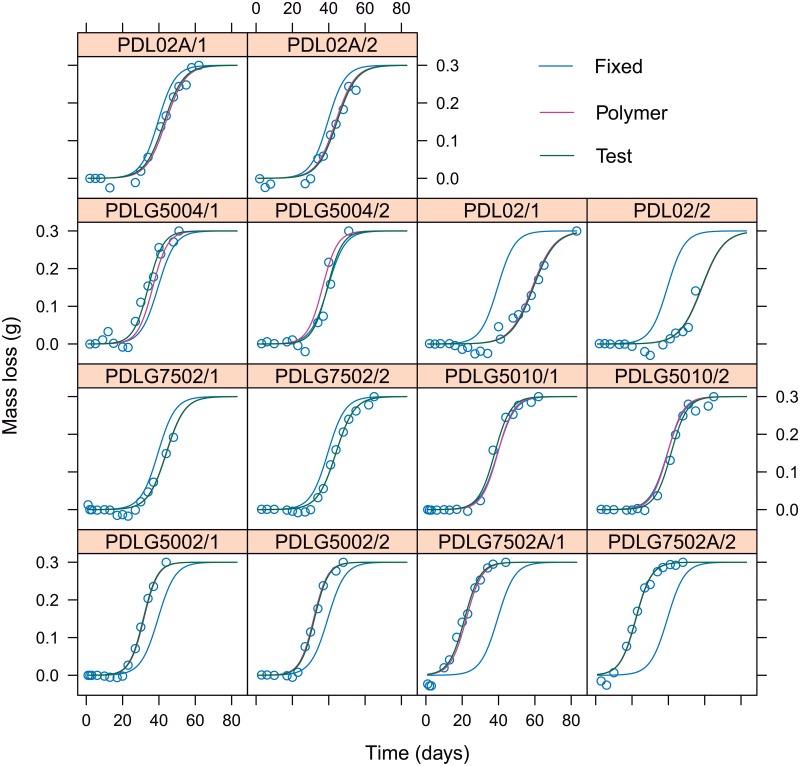
Each panel shows the PDLGA mass loss versus time for each test/replicate corresponding to each PDLGA polymer. The nonlinear mixed-effects fitting for each test is shown and compared with respect to fittings corresponding to each polymer, and the fitting corresponding to the population (and obtained using the fixed effects of model parameters).

The Fig 8 shows the fittings of nonlinear mixed-effects model to estimate mass loss as a function of time, taking into account random effects of PDLGA type and test (replicate) factors. Each panel corresponds to the fitting of the trend corresponding to one replicate of each polymer, shown in dark green. The fitting corresponding to each PDLGA polymer formulation is also shown in pink (it is the same for each PDLGA type), whereas the fitting corresponding to the fixed parameters is shown in blue and accounts for the model adjusted for the entire population of PDLGA formulations (mean degradation path). Table 2 shows the 95% confidence intervals for fixed effects of *ϕ*_3_ and *ϕ*_4_ parameters. We can infer that the half life time of PDLGA polymers is between 31.52 and 47.42 days, while the degradation rate (scale) parameter is between 4.088 and 5.378. Observing Fig 8, the model shows good fittings to real data, preventing to adjust the experimental error, e.g. those corresponding to negative mass loss due to the accuracy degree of the laboratory experimental methodology shown in the Section 2. This is a relevant advantage with respect to nonparametric GAM models.

**Table 2:** Confidence intervals at 95% for fixed effects of the model parameters.

	Lower limit	Estimate	Upper limit
*ϕ*_3_	31.52	39.47	47.42
*ϕ*_4_	4.088	4.733	5.378

**Fig 9:**
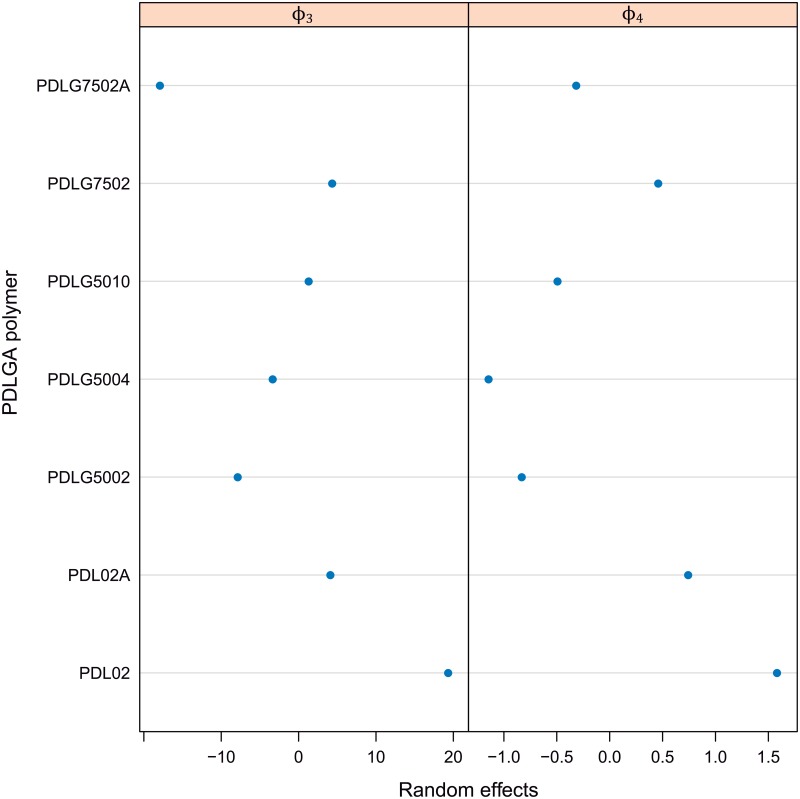
Random effects, *b*_3_*i* and *b*_4_*i* of *ϕ*_3_ and *ϕ*_4_ parameters corresponding to the nonlinear mixed-effect model of mass loss vs. time and PDLGA formulation.

Considering there is not almost differences between the fittings corresponding to the two different tests within each type of PDLGA polymer, and in order to simplify the model, only the PDLGA factor can be assumed. In fact, all the main differences between fitting trends are due to the polymer formulation. The corresponding estimated parametric model (for each *i* polymer) with random effects only due to PDLGA polymer is shown through the expressions 7 and 8. The random effects, *b*_3*i*_ and *b*_4*i*_, for this study case can be also observed in Fig 9.

$$\begin{array}{c} \begin{array}{c} Mass\;Loss_{ij}=\frac{0.3-0}{1+\exp(\frac{Time_{ij}-\phi_{3i}}{\phi_{4i}})}+\epsilon_{ij}\\ \phi_{i}=[\begin{array}{c} \phi_{3i}\\ \phi_{4i} \end{array}]=[\begin{array}{c} \beta_{3}\\ \beta_{4} \end{array}]+[\begin{array}{c} b_{3i}\\ b_{4i} \end{array}]=\beta +b_{i};\;\;b_{i}∼N\left(0,\Psi \right),\;\epsilon_{ij}∼N\left(0,\sigma^{2}\right) \end{array} \end{array}$$ (7)

$$\begin{array}{c} \begin{array}{c} \phi_{i}=[\begin{array}{c} \phi_{3i}\\ \phi_{4i} \end{array}]=[\begin{array}{c} 39.63\\ 4.583 \end{array}]+[\begin{array}{c} b_{3i}\\ b_{4i} \end{array}];\;b_{i}∼N(0,[\begin{array}{cc} 10.74^{2} & 0.0692\\ 0.0692 & 0.9554^{2} \end{array}])\\ \epsilon_{ij}∼N\left(0,0.015^{2}\right) \end{array} \end{array}$$ (8)

Figs 8 and 9 are very useful tools to characterize the hydrolytic degradation of PDLGA biopolymers and comparing them. Fig 8 permits to divide the sample in two groups, those characterized by a degradation path shifted to the left with respect the mean degradation path (in blue) and those defined by a degradation path shifted to the right. Namely, there is a group composed of PDLG7502A, PDLG5002, and PDLG5004 that degrades before than the PDLGA formulations mean, whereas the PDL02, PDL02A, and PDLG7502 are more stable than the mean (the remaining PDLG5010 degradation path coincide just with the mean degradation path). The more stable against hydrolytic degradation seems to be the PDL02, while the less stable is the PDLG7502A. To quantify these differences, the estimated random effects of *ϕ*_3_ and *ϕ*_4_ can be observed (Fig 9). If *b*_3*i*_ is studied, the polymer with a higher half life is clearly the PDL02 (19 days over the population mean *ϕ*_3_), whereas the polymer with a less half life is effectively the PDLG7502A (18 days below the population mean *ϕ*_3_). Moreover, the same conclusions obtained with GAM modelling (Section 2) can be observed. Namely, PDLGA formulations with a higher viscosity midpoint have higher half life: the corresponding to PDLG5010 is 4.6 days higher than the corresponding to PDLG5004, and the PDLG5004 half life is 4.5 days higher than the corresponding to PDLG5002 polymer. On the other hand, the presence of acid terminations decrease the stability against hydrolytic degradation: PDL02 half life is 15 days higher than the corresponding to PDL02A, and the PDLG7502 half life is 22 days higher than the corresponding to PDLG7502A. Moreover, the increment of DL-lactide molar ratio increases the polymer half life: PDLG7502 half life is 12 days higher than the corresponding to PDLG5002. Regarding the random effects of *ϕ*_4_ parameter, *b*_4*i*_, firstly, it is important to note that higher values of *ϕ*_4_ are related to lower degradation rate, and, thus, more degradation stability. Therefore, similar conclusions than in the case of *b*_3*i*_ analysis can be observed. In fact, the addition of acid terminations decreases the *b*_4*i*_ and accordingly increases the rate of degradation (observe the *b*_4*i*_ for PDL02 and PDL02A, and PDLG7502 and PDLG7502A). The same trend is obtained when decreasing the DL-lactide molar ratio (see *b*_4*i*_ for PDLG7502 and PDLG5002). The highest rate of degradation (lowest *b*_4*i*_) are obtained for PDLG5002 and PDLG5004. The increment of midpoint viscosity to 10 dl/g significantly decreases the rate of degradation. Concluding, nonlinear mixed-effects modelling provide very usefull information to characterize the degradation paths of the PDLGA biopolymers. The provided information can help to develop selection criteria of these materials with respect the application as dental scaffolds.

**Fig 10:**
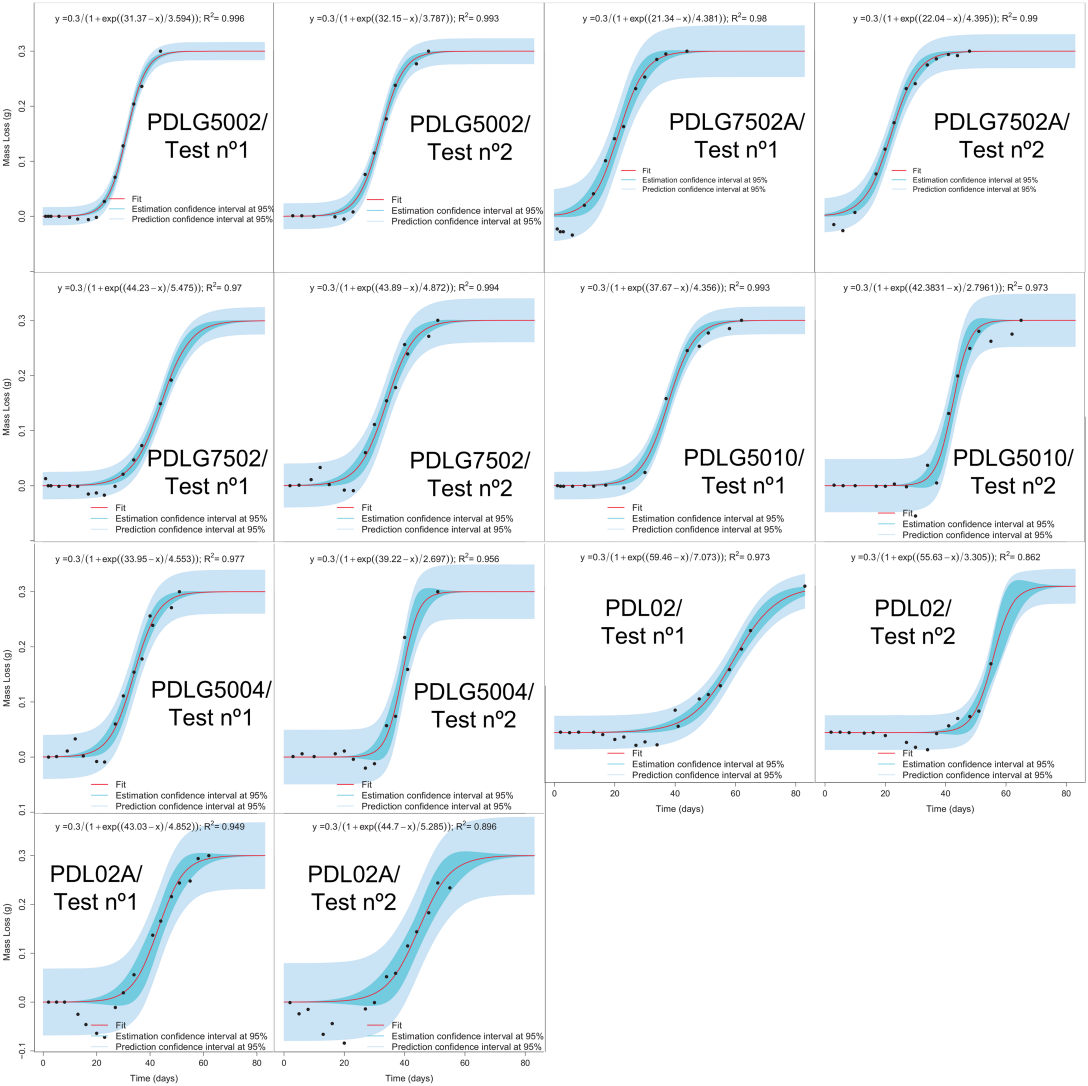
The fittings of fixed effects nonlinear regression models based on logistic function are shown. Each panel corresponds with the fitting of each degradation path. The expression of the model (with parameter estimates), goodness of fit measurements (R^2^) and both prediction and estimation intervals are also shown.

The results of the application of GAM models and mixed effects nonlinear regression models have shown that the degradation paths are different depending on the polymer formulation. In fact, for instance, the Fig 8 shows and compares the fits obtained from (1) all the samples (the mean degradation trend of PDLGA polymers, tagged as “fixed”), from (2) each PDLGA formulation (tagged as “polymer”), and from (3) each replicate (out of two) corresponding to each PDLGA formulation (tagged as “test”). In addition, for the sake of clarity, fixed effects nonlinear regression models based on logistic function have been fitted to the mass loss vs time relationship. The fitting of nonlinear regression models have been performed by applying (1) evolutionary algorithms in order to obtain an initial solution for the parameters, and subsequently (2) the Levenberg-Marquardt Nonlinear Least-Squares Algorithm to obtain the final parameter estimates, as shown in Janeiro-Arocas et al. [45]. These models have been applied separately to each degradation path (out of two) corresponding to each polymer, obtaining the results shown in the Fig 10. It is important to note that all the parameters are significantly different from zero. Moreover, the goodness of fit is very high in all the cases (R^2^ > 0.86, and in the main part of PDLGA polymers RR^2^ > 0.95). Those cases where the goodness of fit is lower are mostly related to the fact that the proposed models do not fit the experimental error (laboratory error) corresponding to negative mass loss, as expected. Fig 10 shows there are mostly slightly differences between the fitting parameters corresponding to the two different tests or replications of each PDLGA polymer. However, there are higher differences between the parameters fitted to different polymers. The results are in accordance with the results corresponding to GAM and mixed effect models. The interpretation of the parameter estimates is also in accordance with the discussion corresponding to the mixed effect nonlinear models. When the study of these specific 7 PDLGA polymers is intended (more than the degradation trend of PDLGA population), the present fixed nonlinear regression approach provides a more intuitive and clear interpretation of PDLGA polymers degradation.

**Fig 11:**
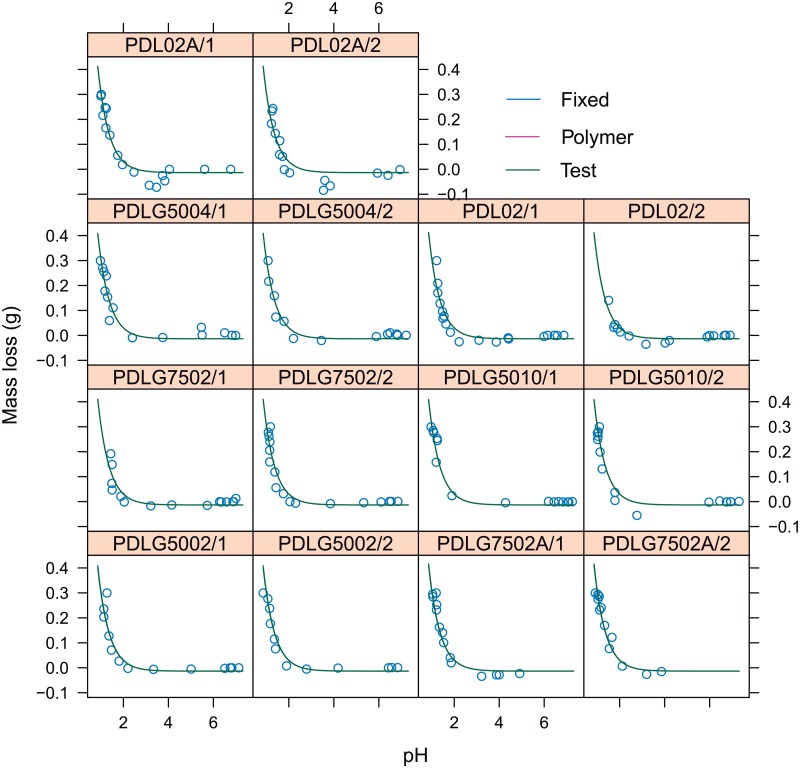
Each panel shows the PDLGA mass loss versus pH for each test/replicate nested in PDLGA polymer factor. The nonlinear mixed-effects fitting for each test is shown and compared with respect to fittings corresponding to each polymer, and the fitting corresponding to the population (and obtained using the fixed effects of model parameters).

**Fig 12:**
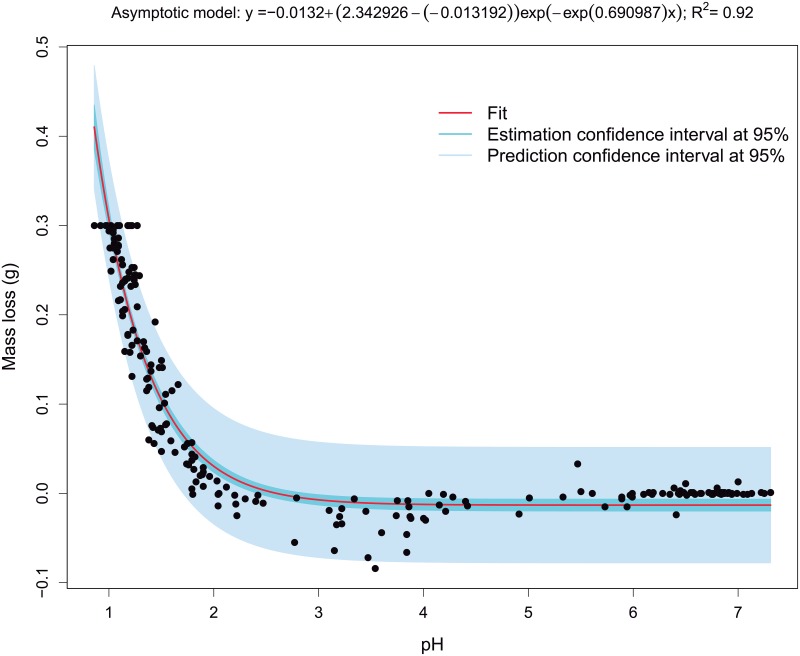
Asymptotic fixed effects regression model fitted mass loss vs. pH data. Fitted trend, real data, 95% estimation and prediction confidence intervals for conditional mean are included.

The second important relationship to be modelled is the corresponding to the mass loss versus polymer pH. Nonlinear mixed-effects model based on asymptotic function, with random effects in all the parameters, is initially proposed taking into account the results of exploratory analysis and GAM modelling. Fig 11 shows the graphical output corresponding to the nonlinear mixed-effect model, in which panel the fittings corresponding to each test (in dark green), the PDLGA polymer (in pink) and the PDLGA population (in blue, obtained from the fixed effects of model parameters). Intuitively, exact and accurate fittings have been obtained, preventing to fit the experimental error of negative mass loss. Thus, the asymptotic function is suitable to model this relationship. It is also very important to stress that the same fit has been obtained for test sample, PDLGA polymer and population in each panel. This means that mass loss does not depend on PDLGA polymer formulation and test/replication factor. Therefore, the application of nonlinear fixed effects regression modelling is recommended. Fig 12 shows the result of asymptotic nonlinear regression model with only fixed effects. The model parameters and signification analysis (all the parameters are significant different from zero, *p*-values<0.05) are shown in Table 3. Taking into account that the *ϕ*_3_ is the degradation constant logarithm, the half life is 0.691, i.e. the pH at which the half of mass is loss. The polymer pH explain the 92% of the mass loss overall variability (*R*^2^ = 0.92). Hence, the proposed asymptotic model could provide reliable estimations for mass loss only knowing the pH. As pointed out by Schliecker et al. [30], this fact could be related to the possible formation of free carboxylic acid groups during the PDLGA degradation, increasing its concentration due to an autocatalytic process, and leading to an asymptotic (in the present case) decrement of the polymer pH from 7.4. Thus, the polymer pH could be an index of the amount of carboxylic acid, in turn related to the degree of degradation of PDLGA polymer. This could be due to water soluble hydrolytic degradation products with low molecular weight may remain entrapped, increasing the concentration of carboxylic end group (decreasing the pH) [30]. This conditions in which the degradation products remain in contact with the non-degraded mass may correspond to the experimental study carried out in this work.

**Table 3:** Signification analysis of the regression model for the mass loss according to the pH for each PDLGA polymer (*R*^2^ = 0.92).

	Estimates	Standard deviation	t values	*p*-values
*ϕ*_1_	-0.013	0.003	-3.909	0.0001
*ϕ*_2_	2.343	0.253	9.248	0
*ϕ*_3_	0.691	0.049	14.06	0

**Fig 13:**
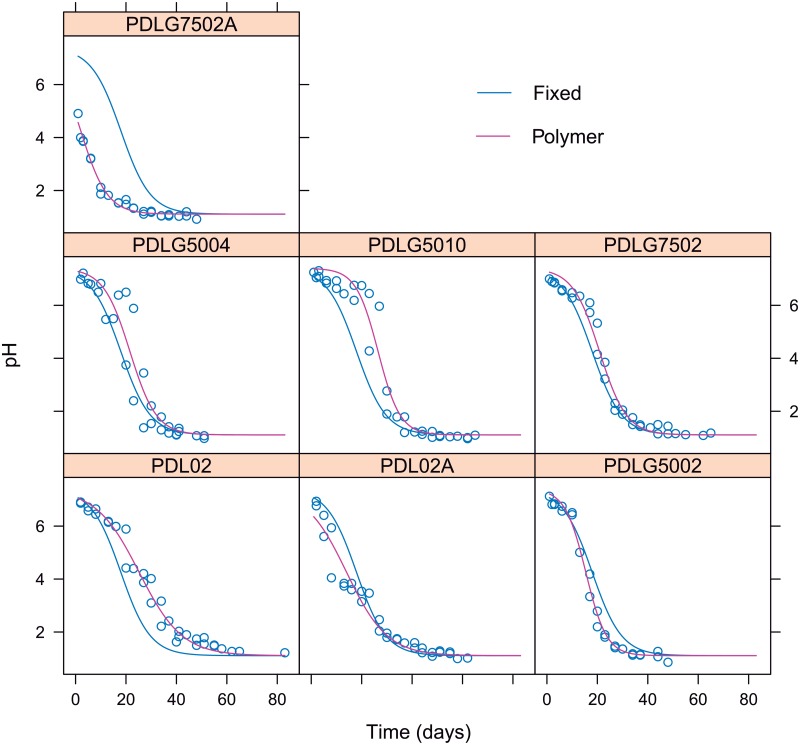
Each panel shows the PDLGA pH versus time for each PDLGA polymer. The nonlinear mixed-effects fitting for each PDLGA polymer is shown and compared with respect to fittings corresponding to the population (obtained using the fixed effects of model parameters).

**Fig 14:**
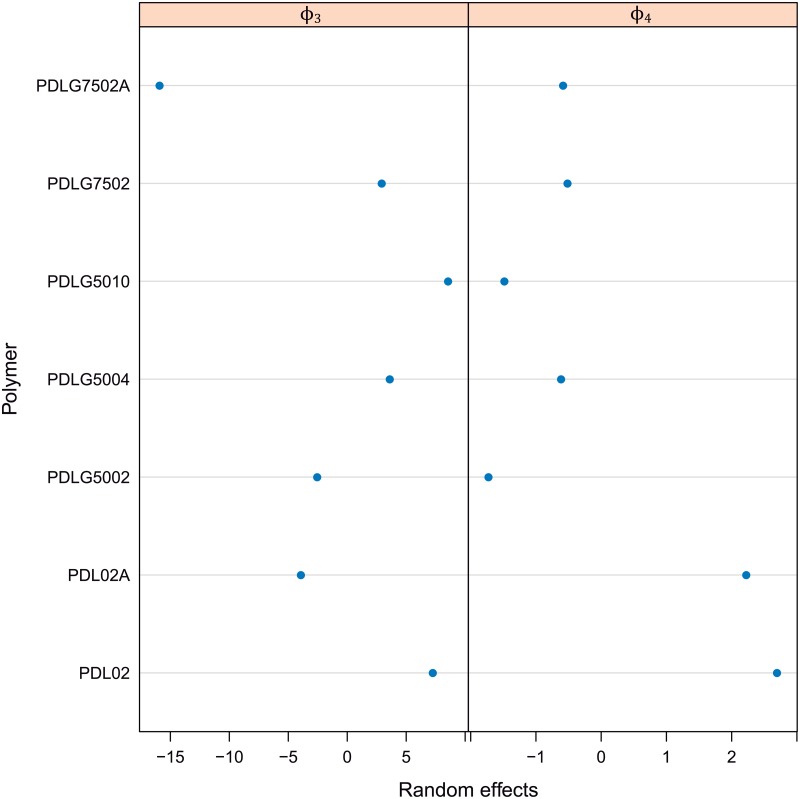
Random effects, *b*_3*i*_ and *b*_4*i*_ of *ϕ*_3_ and *ϕ*_4_ parameters corresponding to the nonlinear mixed-effect model of pH vs. time and PDLGA formulation.

On the basis of PDLGA mass loss is a critical variable to evaluate the degree of hydrolytic degradation [30] and that this can be accurately estimated knowing the pH through the estimation of an asymptotic regression function, hence we should ask about what is the cause of which the mass loss trend is different depending on the PDLGA formulation. The answer is that pH decays, as a function on time, in a different manner depending on PDLGA polymer. In fact, nonlinear mixed-effects regression model based on a decreasing logistic function can successfully fit all the trends corresponding to each out of two tests corresponding to each PDLGA polymer (see Fig 13). Random effects on *ϕ*_2_ and *ϕ*_3_ are assumed and an initial asymptote fixed to the initial pH = 7.4 (Fig 14). The more stable biopolymers are those whose pH decays later and with a lower rate. Accordingly with Figs 13 and 14, taking into account the balance between half life (*ϕ*_3_) and degradation rate (*ϕ*_4_), the more stable polymer against hydrolytic degradation are PDL02, PDL02A, PDLG510, and PDLG7502, whereas the less stable are PDLG7502A, PDLG5002, and PDLG504. Although the half life of PDL02A is lower than the corresponding to PDLG5002, its degradation rate is very low, thus PDL02A takes longer to degrade. These results are in accordance with the conclusions corresponding to the mass loss vs. time and PDLGA formulation modelling. It is also important to highlight that half life is decreasing when acid termination is included but the degradation rate nearly remain the same (Fig 14). When increasing the molar fraction of DL-lactide monomer, the degradation rate decrease and the half life increase. Moreover, increasing midpoint viscosity produces analogous increasing in half life (no effect is observed in the degradation rate). With respect to degradation rate (or rate of pH decay), this is lower for PDL polymers with respect to the studied PDLGA copolymers.

The fitted nonlinear mixed-effects model can be writen for each polymer as

$$\begin{array}{c} \begin{array}{c} pH_{i}=7.4+\frac{\phi_{2i}-7.4}{1+\exp(\frac{Time_{ij}-\phi_{3i}}{\phi_{4i}})}+\epsilon_{ij}\\ \phi_{i}=[\begin{array}{c} \phi_{2i}\\ \phi_{3i}\\ \phi_{4i} \end{array}]=[\begin{array}{c} 0.9946\\ 12.12\\ 4.477 \end{array}]+[\begin{array}{c} b_{2i}\\ b_{3i}\\ b_{4i} \end{array}]=b_{i}∼N(0,[\begin{array}{ccc} 0 & 0 & 0\\ 0 & 7.793^{2} & 0.0062\\ 0 & 0.0062 & 1.714^{2} \end{array}])\\ \epsilon_{ij}∼N\left(0,0.4485^{2}\right) \end{array} \end{array}$$ (9)

The GAM and nonlinear regression tools applied in this work have been proven useful to characterize and compare PDLGA biopolimers with application as dental scaffolds, helping to develop selection criteria, previously to perform in vivo analysis.

Finally, in order to estimate statistically the effects of of DL-lactide molar ratio, the viscosity, the addition of acid termination and the corresponding interactions, an ANOVA model with scalar response is fitted. The aim is to support the conclusions obtained by the application of the above mentioned GAM and nonlinear regression models. It is important to stress that the response variable is functional (each datum is a curve) as defined in this work. Thus, in order to be able to apply ANOVA tools for univariate response, the authors have defined as response variable the mass loss at 34 days from the beginning of the experiment. The relationship between mass loss and time is nonlinear sigmoid, but could be assumed linear in the central interval of times where Time = 34 days is included. The Table 4 shows that the effects of acid termination, DL-lactide molar ratio and viscosity are significantly different from zero. These results help to support the discussion on the effect of these factors from the application of GAM and nonlinear regression modelling. Moreover, the there is a significant effect of the interaction of the acid termination with the molar ratio over the mass loss response.

**Table 4:** ANOVA table with F test. Only the factors with significant effects over the response (mass loss) are shown.

	Sum of Squares	Degrees of freedom	F values	*p*-values
Acid Termination	0.052650	1	59.7222	0.0002464
Molar Ratio	0.089664	2	50.8541	0.0001729
Viscosity	0.017082	2	9.6881	0.0132181
Acid Termination:Molar Ratio	0.012246	1	13.8911	0.0097704
Residuals	0.005289	6		

In addition, considering the effect of time over mass loss is nonlinear sigmoid, a generalized additive regression model (GAM) with mass loss as response variable, time as quantitative predictor, and DL-lactide molar ratio, the viscosity, and the addition of acid termination as qualitative independent variables is also proposed. In this way, the effect of the latter factors and their interactions can be studied apart from the nonlinear effect of time over the response, in the same way the standard ANCOVA analysis is performed when the effect of covariates are linear. Table 5 shows that the mass loss significantly decreases when the viscosity is increasing to 10 dl/g, and when the DL-lactide molar ratio is increased (see estimates and p-values columns). Otherwise, the mass loss significantly increases when acid termination is included. When the nonlinear effect of time covariate is included through this model, the effect of interactions does not make the model more informative, in terms of goodness of fit and signification analysis. Moreover, as expected, the effect of time is significantly different from zero. All these results support and complete the above mentioned discussion.

**Table 5:** GAM estimates and signification study corresponding to the modelling of mass loss vs time (quantitative), acid termination (factor), DL-lactide molar ratio (factor), and viscosity (factor). The measure of goodnes of fit is R^2^ = 0.8.

	Estimates	Std. error	t value	*p*-values
Intercept	0.118566	0.010376	11.426	<2e-16
Viscosity = 4	-0.019618	0.014307	-1.371	0.17184
Viscosity = 10	-0.038887	0.014063	-2.765	0.00622
Molar Ratio = 75%	-0.038143	0.013229	-2.905	0.00408
Molar Ratio = 100%	-0.128157	0.013289	-9.644	<2e-16
Acid termination = YES	0.080034	0.009473	8.448	5.95e-15
	EDF	Ref. degrees of freedom	F	p-value
s(Time)	4.266	5.289	136.5	<2e-16

## 5 Conclusions

In the present work, the application of statistical modelling, based on GAM and nonlinear mixed effects regression, to obtain information of PDLGA biopolymers degradation has been proposed. Namely, the critical variables that best describe the hydrolytic degradation process of the studied PDLGA polymers have been identified, their dependence relationships have been defined by applying statistical modelling, and the different PDLGA formulations have been compared by using the models estimates in order to help to develop selection criteria in dental scaffolding and other medical applications before in vivo analysis.

Firstly, the statistical descriptive analysis and GAM modelling have provided information about critical variables for PDLGA polymers degradation and their pairwise dependence relationships. In fact, the relationship between mass loss and time is increasing sigmoid, the effect of pH over the mass loss is decreasing asymptotic, the effect of *T*_*g*_ over mass loss is decreasing sigmoid, whereas the effect of the PDLGA formulation is significant different from zero and different depending on the formulation. It is very important to stress that, reliable in vitro hydrolytic degradation degree predictions could be obtained using GAM models with pH as independent variable, considering that the dependence relationship between mass loss and pH is very strong and the 94% of mass loss variability is explained by the smooth effect of pH (*R*^2^ = 0.939). Alike, the mass loss of PDLGA polymers (degree of degradation feature) can be also estimated using GAM models as a function of time and PDLGA formulation (*R*^2^ = 0.802).

Considering these results, developing parametric models with physical-chemical meaning has been proven a helpful tool to compare different formulations in addition to predict their degradation degree. Nonlinear mixed-effects nonlinear regression is proposed to perform these tasks taking into account the nonlinear dependence relationships between critical variables and the fact data are grouped according to PDLGA formulation and replicate factors. At this regard, the critical variable for describing the degradation degree, mass loss, has been modelled as a nonlinear increasing logistic function depending on time, including fixed and random effects in the logistic parameters. As a result, the half life time of studied PDLGA polymers is between 31.52 and 47.42 days, while the degradation rate parameter is between 4.088 and 5.378. Random effects corresponding to PDLGA polymer formulation have been included in the half life (*ϕ*_3_) and degradation rate (*ϕ*_4_) parameters, allowing us to compare the different PDLGA biopolymers. Consequently, two groups of PDLGA polymers have been identified: a set composed of PDLG7502A, PDLG5002, and PDLG5004 that degrade before than the PDLGA formulations mean, and a second group composed of the PDL02, PDL02A, and PDLG7502 polymers that are more stable than the mean (PDLG5010 degradation path coincides with the mean degradation path). If random effect (*b*_3*i*_) of half life (*ϕ*_3_) is studied, the polymer with a higher half life against hydrolytic degradation is the PDL02 (19 days more than the population mean *ϕ*_3_), while the biopolymer with a less half life expectancy is the PDLG7502A (18 days below the population mean *ϕ*_3_). The same conclusions are obtained if the random effect *b*_4*i*_, related to the polymer rate of degradation, is studied. It is important to note that the differences of in vitro hydrolytic degradation stability of biopolymers can be quantified through the proposed statistical modelling proposal, allowing to develop selection criteria. Moreover, nonlinear mixed-effects modelling provides information about the influence on degradation stability of PDLGA characteristics such as the presence of acid terminations, the midpoint viscosity, the DL-lactide molar ratio and the copolymer structure. Namely, PDLGA formulations with a higher viscosity midpoint tend to have higher half life and lower rate of degradation. In addition, the presence of acid terminations decreases the stability against hydrolytic degradation (in terms of half life and rate of degradation), whereas the increment of DL-lactide molar ratio increases the polymer half life (and decreases the rate of degradation). These conclusions are also supported by the results of statistical nonparametric GAM modelling and ANOVA.

It is important to highlight that mass loss can be accurately determined by a nonlinear asymptotic function of polymer pH, independently of PDLGA polymer formulation. Accordingly, a nonlinear asymptotic model has been estimated to describe the dependence relationship between mass loss and pH, explaining the 92% of the mass loss overall variability (*R*^2^ = 0.92), also obtaining that the half of the mass is loss at a pH of 0.691. This strong relationship could be related to the formation of free carboxylic acid groups during the PDLGA degradation that increase their concentration due to water soluble hydrolytic degradation products with low molecular weight may remain entrapped. Therefore, the polymer pH can be assumed as an index of the amount of carboxylic acid, and thus related to the degradation degree of PDLGA polymers.

The mass loss trend is different depending on the PDLGA formulation due to the PDLGA pH decays according to different time depended logistic functions for each biopolymer formulation. Hence, nonlinear mixed-effects regression model based on a decreasing logistic function has been fitted to describe the dependence relationship between pH and time. The estimated parameters have been analyzed, obtaining conclusions in accordance with the obtained for the mass loss vs time relationship study. Moreover, it is also important to note that, although half life is decreasing when acid termination is included, the degradation rate does not change. The same effect is obtained when increasing midpoint viscosity. In addition, the rate of pH decay (related to the degradation rate) is lower for PDL polymers with respect to the PDLGA counterparts.

The proposed methodologies could be applied to other biopolymers from in vitro test data in order to identify critical variables for hydrolytic degradation, estimate the degradation path of each biopolymer, predict its behaviour and to help to develop selection criteria.

## Supporting information

S1 Dataset Dataset obtained from experimental analysis. The polymer mass loss, the mass of water, the molecular weight, the glass transition temperature and the pH variables have been monitored depending on time, for all the studied polymer formulations, obtaining two replicates (test variable) per polymer and time. The viscosity, DL-lactide molar ratio, and presence of acid term variables corresponding to each polymer formulation are also included in the dataset.
(TXT)
