# Correction: Statistical degradation modelling of Poly(D,L-lactide-co-glycolide) copolymers for bioscaffold applications

**DOI:** 10.1371/journal.pone.0206833

**Published:** 2018-10-29

**Authors:** Yaroslava Robles-Bykbaev, Javier Tarrío-Saavedra, Sara Quintana-Pita, Silvia Díaz-Prado, Francisco Javier García Sabán, Salvador Naya

The following information is missing from the Funding section: The research of Salvador Naya and Javier Tarrío Saavedra has also been supported by MINECO [Grant MTM2017-82724-R].

## References

[1] Robles-Bykbaev Y, Tarrío-Saavedra J, Quintana-Pita S, Díaz-Prado S, García Sabán FJ, Naya S (2018) Statistical degradation modelling of Poly(D,L-lactide-co-glycolide) copolymers for bioscaffold applications. PLoS ONE 13(10): e0204004 10.1371/journal.pone.0204004 30273349 PMC6166939

